# Detection of Microsatellite Instability in Colonoscopic Biopsies and Postal Urine Samples from Lynch Syndrome Cancer Patients Using a Multiplex PCR Assay

**DOI:** 10.3390/cancers14153838

**Published:** 2022-08-08

**Authors:** Rachel Phelps, Richard Gallon, Christine Hayes, Eli Glover, Philip Gibson, Ibrahim Edidi, Tom Lee, Sarah Mills, Adam Shaw, Rakesh Heer, Angela Ralte, Ciaron McAnulty, Mauro Santibanez-Koref, John Burn, Michael S. Jackson

**Affiliations:** 1 Biosciences Research Institute, Faculty of Medical Sciences, Newcastle University, Newcastle upon Tyne NE2 4HH, UK; 2 Translational and Clinical Research Institute, Faculty of Medical Sciences, Newcastle University, Newcastle upon Tyne NE2 4HH, UK; 3 Department of Histopathology, Northumbria Healthcare NHS Foundation Trust, Newcastle upon Tyne NE27 0QJ, UK; 4 Department of Gastroenterology, Northumbria Healthcare NHS Foundation Trust, Newcastle upon Tyne NE27 0QJ, UK; 5 Population Health Sciences Institute, Faculty of Medical Sciences, Newcastle University, Newcastle upon Tyne NE2 4AX, UK; 6 Department of Surgery, Northumbria Healthcare NHS Foundation Trust, Newcastle upon Tyne NE27 0QJ, UK; 7 Guy’s & St Thomas’ NHS Foundation Trust, Great Maze Pond, London SE1 9RT, UK; 8 South of Tyne and Wear Pathology and Northern Gynaecological Oncology Centre, Queen Elizabeth Hospital, Gateshead Health NHS Trust, Gateshead NE9 6SX, UK; 9 The Newcastle upon Tyne Hospitals NHS Foundation Trust, Newcastle upon Tyne NE7 7DN, UK

**Keywords:** Lynch syndrome, mismatch-repair deficiency, microsatellite instability, tumour biopsy, liquid biopsy

## Abstract

**Simple Summary:**

Lynch syndrome is caused by inherited defects in genes which repair DNA, and predisposes affected individuals to cancers of the bowel, endometrium (uterus lining), and urinary tract, among others. Most of these cancers exhibit a characteristic pattern of DNA instability in Lynch syndrome patients. Here, we adapt an existing DNA instability test for use with small clinical samples, and show that it works with tumour biopsies taken during colonoscopic investigation just as effectively as with larger samples taken during surgery. We also analyse preoperative postal urine samples where DNA recovery is limited, identifying instability in samples from a Lynch syndrome patient with a urinary tract cancer, and a patient with a suspected endometrial cancer. The assay could enable earlier detection of these tumours, benefiting these patients.

**Abstract:**

Identification of mismatch repair (MMR)-deficient colorectal cancers (CRCs) is recommended for Lynch syndrome (LS) screening, and supports targeting of immune checkpoint inhibitors. Microsatellite instability (MSI) analysis is commonly used to test for MMR deficiency. Testing biopsies prior to tumour resection can inform surgical and therapeutic decisions, but can be limited by DNA quantity. MSI analysis of voided urine could also provide much needed surveillance for genitourinary tract cancers in LS. Here, we reconfigure an existing molecular inversion probe-based MSI and *BRAF* c.1799T > A assay to a multiplex PCR (mPCR) format, and demonstrate that it can sample >140 unique molecules per marker from <1 ng of DNA and classify CRCs with 96–100% sensitivity and specificity. We also show that it can detect increased MSI within individual and composite CRC biopsies from LS patients, and within preoperative urine cell free DNA (cfDNA) from two LS patients, one with an upper tract urothelial cancer, the other an undiagnosed endometrial cancer. Approximately 60–70% of the urine cfDNAs were tumour-derived. Our results suggest that mPCR sequence-based analysis of MSI and mutation hotspots in CRC biopsies could facilitate presurgery decision making, and could enable postal-based screening for urinary tract and endometrial tumours in LS patients.

## 1. Introduction

Lynch syndrome (LS) is an autosomal-dominant cancer syndrome caused by germline variants in 1 of 4 DNA mismatch repair (MMR) genes: mutL homologue 1 (*MLH1*), mutS homologue 2 (*MSH2*), mutS homologue 6 (*MSH6*), and much less frequently PMS1 homologue 2 (*PMS2*) [1,2]. LS is estimated to affect 1 in 279 people [3], and predisposes to a range of malignancies, most notably colorectal cancer (CRC) and endometrial cancer (EC), with the cumulative risk of any cancer reaching ~75% for *MLH1*-variant carriers and ~85% for *MSH2*-variant carriers by the age of 75 years [2]. MMR ensures the fidelity of DNA replication [4], and MMR deficiency (MMRd) leads to hypermutability and microsatellite instability (MSI), a molecular phenotype caused by failure to repair polymerase slippage-induced indels in short tandem repeats [5].

Once known to the health-care system, LS patients can benefit from tumour surveillance measures, such as colonoscopy, and aspirin chemoprophylaxis, which can reduce future tumour incidence [6,7]. However, only 5–10% of an estimated 175,000 affected individuals in the UK have been identified to date [8]. In 2017, the UK National Institute for Health and Care Excellence (NICE) introduced recommendations that LS testing be offered to all CRC patients (https://www.nice.org.uk/guidance/dg27 accessed on 30 May 2022), but these have yet to be fully implemented; analysis of all colorectal cancer testing across England in 2019 has revealed a range from 16% to 70% (mean 42%) for first-stage analysis (National Disease Registration Service, NHS England, paper in preparation). Testing of endometrial cancers has since been advocated [9] and adopted within NICE diagnostics guidance (https://www.nice.org.uk/guidance/dg42 accessed on 30 May 2022).

For the majority of LS patients, the diagnostic pathway begins with analysis of resected CRC material, either through analysis of all 4 MMR proteins by immunohistochemistry (IHC) or analysis of MSI within tumour DNA. MMRd tumours are then tested further to eliminate those where instability is somatic in origin, before screening for germline pathogenic MMR variants (https://www.nice.org.uk/guidance/dg27/resources/testing-strategies-flowchart-pdf-4367005453 accessed on 30 May 2022). Knowledge of tumour MMR status is also important because it can influence treatment; the adjuvant 5-flurouracil confers no survival benefit in MMR-deficient CRC [10,11], MMR-deficient metastatic CRC responds much better to capecitabine and oxaliplatin [12], and MMR-deficient tumours respond better to the programmed cell death protein 1 (PD-1) inhibitor pembrolizumab [13,14].

Establishing tumour MMR status earlier in the diagnostic pathway could accelerate LS detection and enhance patient care. A number of recent analyses suggest that the application of machine learning to computed tomography [15,16,17] or infrared [18,19,20] imaging may have the potential to discriminate microsatellite stable (MSS) from microsatellite unstable (MSI-High/MSI-H) tumours. However, such methods are not routinely available to all patients, and have many competing demands for their use. Analysis of colonoscopic biopsies currently represents a more viable approach, and would still facilitate LS diagnosis prior to surgery and allow more extensive resection to be considered to reduce the risk of metachronous tumours [21,22]. There is high concordance between the MMR status of colonoscopic biopsies assayed using IHC and resected tumour material [23,24,25,26,27,28]. IHC is a low-throughput method requiring expert interpretation, but a wide variety of MSI assays are available, including microsatellite fragment-length analysis, analysis of microsatellites within exome or whole genome sequence, targeted amplicon sequencing, qPCR, and digital droplet PCR (reviewed in [29,30]). These vary in cost, turnaround time, throughput, and ease of integration with other elements of LS-detection pathways.

Amplicon sequencing-based MSI assays may be particularly suitable for biopsy analysis, as they are cheap, easy to scale and automate, and can incorporate detection of actionable mutations. We have previously developed a molecular inversion probe (MIP)-based amplicon sequencing assay with 24 mononucleotide markers, and shown that as few as 6 give complete discrimination between MSS and MSI-H CRCs. The assay requires as little as 3% MSI-H DNA, incorporates *BRAF* c.1799T > A p.(V600E) detection to streamline LS screening [31], and is sensitive enough to detect low-level MSI in non-neoplastic soma of individuals with constitutional mismatch-repair deficiency (CMMRD [32]).

Here, we investigate whether this MSI assay could be used to detect MMR-deficient CRCs from colonoscopic biopsies. We reconfigure the assay to a 12-marker multiplex PCR (mPCR) format suitable for use with low template quantities, and analyse both individual and composite biopsies from LS patients with CRC. As mPCR can be used to screen for gene-specific mutations in cell-free DNA (cfDNA) from urine samples [33,34], we also assess the assay’s ability to identify MSI in cfDNA. This is of particular interest in the context of LS, as upper tract urothelial cancer (UTUC) is the 3rd most common LS malignancy [35] and has significantly lower 10-year survival than colon cancer [36]. LS patients are more likely to die as a result of UTUC than CRC [36], and *MSH2*-variant carriers are at particular risk, with a >20% chance of developing a UTUC by the age of 75 [2]. There is no routine surveillance for this tumour type in LS, but as 75–98% of LS-derived UTUCs are MMRd [29], MSI is a potentially useful cancer biomarker. There is also an unmet clinical need for early, non-invasive detection of endometrial cancer (EC) [37], the 2nd most common LS malignancy. A number of biomarkers for EC have been investigated in patient urine [38], and urine/vaginal cytology has recently been shown to identify gynaecological cancers with high sensitivity and specificity (91.7% and 88.8%, respectively [39]). As proof of principle, we therefore also analyse preoperative (preop) and postoperative (postop) urine samples from two LS patients, one with a confirmed UTUC, the other a suspected EC.

## 2. Materials and Methods

### 2.1. Patient Samples

Colonoscopic biopsies from 4 LS CRC patients were obtained from the Northumbria Health Care NHS Foundation Trust following a review of patient data and sample availability carried out using the Telepath Laboratory Information Management System and a Hamamatsu S210 slide scanner. Ten micron curls were taken as “composite” samples from the FFPE tumour block containing all biopsies from each patient. The block was then scored to allow collection of curls from each individual biopsy.

The following samples were obtained from a 68-year-old male LS patient with an *MSH2* variant enrolled on the CaPP3 clinical trial (ISRCTN16261285), who was diagnosed with an UTUC in the pelvis of the right kidney and underwent a nephroureterectomy (Case 1): peripheral blood leukocyte (PBL) genomic DNA (gDNA), preop urine, and blood taken 4 weeks prior to surgery (1 month after diagnosis), tumour taken at surgery, postop urine taken 4 weeks after surgery, and postop urine and blood taken 10 weeks after surgery.

The following samples were obtained from a 38-year-old female LS patient with an *MLH1* variant who underwent a hysteroscopy followed by a hysterectomy and bilateral salpingo-oophorectomy (Case 2): urine and vaginal swab samples taken 1 month prior to hysteroscopy, a tumour biopsy taken at hysteroscopy, a further urine sample taken after hysteroscopy, resected tumour, ovary, fallopian tube, uterine wall, and normal endometrium samples taken at hysterectomy/oophorectomy, and 2 postop urine samples taken a further 2 and 3 weeks later.

Voided urine samples were collected in 50 mL falcon tubes containing cell-free DNA urine preservative (Streck, La Vista, NE, USA), or using Colli-Pee (Novosanis, Wijnegem, Belgium) collection tubes with UCM preservative, according to the manufacturer’s recommendations. All preop and postop urine samples were self-collected by patients and delivered by hand or post. Blood was collected in Cyto-Chex BCT (Streck) blood-collection tubes.

Discarded blood samples from 3 non-cancer referrals (controls), and discarded 10-micron formalin-fixed paraffin-embedded (FFPE) tissue curls from 151 resected CRC samples of known MSI status were obtained from the Northern Genetics Service, Newcastle upon Tyne Hospitals NHS Foundation Trust (NuTH), and were used for assay development, training, and testing. Ten resected urothelial cancer (UC) samples from LS patients enrolled on the CaPP3 clinical trial (obtained as ten-micron formalin-fixed paraffin-embedded (FFPE) tissue curls), and 33 preop urine samples from unselected urothelial UC patients treated within the NuTH were also analysed.

### 2.2. DNA Isolation

PBL gDNA was isolated using the Gentra Puregene Blood Kit (Qiagen, Hilden, Germany), gDNA from FFPE tissue was isolated using the GeneRead DNA FFPE Kit (Qiagen), and cfDNA was isolated from urine and plasma using the QiAmp Circulating Nucleic Acid Kit (Qiagen), all in accordance with the manufacturer’s recommended protocols. DNAs were quantified using a Qubit dsDNA HS Assay Kit and QuBit 3.0 fluorometer (Invitrogen, Waltham, MA, USA).

### 2.3. Two-Step Multiplex PCR and MIP Amplification

mPCR primers were designed with 8N molecular barcodes (4N in each primer) to amplify 12 of the 24 mononucleotide repeat markers described by Gallon et al. [31], using PCRTiler v1.42 [40] with GrCH37/hg19 as reference and a melting temperature range of 57–61 °C. These included the 6 most sensitive markers. To maximise amplification efficiency with low-quantity/quality templates, amplicon size was initially set at a maximum of 90 bp, and then increased by 10 bp incrementally if no usable primer pairs were obtained. Primers to identify *BRAF* c.1799T > A p.(V600E) mutations [31], associated with sporadic MMRd tumours, were also designed. Multiplex Manager [41] was used to select primers which minimised primer interactions within the multiplex (see Table S1).

Markers were amplified following the two-step multiplex PCR assay of [42] with the following modifications: the Qiagen Multiplex PCR Kit was used for the first round of PCR, Q5 Hot Start High-Fidelity 2× Master Mix (New England BioLabs, Ipswich, MA, USA, cat. no. M0494S) was used in the second, and an Ampure bead clean-up was introduced between the first and second round of PCR [43] instead of nuclease treatment.

SmMIP-based amplification was conducted following the protocol of [44] and modified by [31]. To facilitate comparative analysis, single-molecule molecular inversion probes (smMIPs) for all MSI markers were also redesigned to include 8N molecular barcodes and give products of the same size range as mPCR (Table S1). mPCR sampled a higher proportion of template molecules than MIPs across a range of control PBL DNA template quantities (30 ng to < 1 ng, Figure S1). Although there was variation in read depth between markers, all were represented in the multiplex (Figure S2). The molecular barcode (MB) count remained above 75 per marker at all template quantities analysed (Figure S3), a previously defined threshold required for accurate MIP-based classification [31]. The mPCR background sequencing error rate was higher than in the parent MIP assay for all markers (Figure S4), both before and after error correction using smSequences (sequences that represent the majority of reads associated with each unique MB, see Section 2.4). However, the corrected mPCR error rate was comparable to MIP raw reads which are used for MSI classification with the 24-marker MIP assay, with median variant allele frequencies (VAFs) of 1.47% and 1.61%, respectively.

### 2.4. Sequencing and MSI Analysis

Amplicons from both assays were visualised using 3% agarose gel electrophoresis, purified using Agencourt AMPure XP Beads (Beckman Coulter, Brea, CA, USA), then pooled and sequenced on a MiSeq platform (Illumina, San Diego, CA, USA) to a target depth of 5000 reads per marker using custom primers [44]. Reads were aligned to GrCH37/hg19 using BWA v0.6.2 [45]. SAM files were analysed using custom R scripts to count reads of different microsatellite allele lengths for each marker, as described previously [31,46]. SAM files were also analysed to count molecular barcodes (MBs) as an estimate of the number of unique template DNA molecules sequenced [31]. The fraction of available templates sampled could then be calculated by dividing by the total number of templates in each reaction, estimated assuming a C-value of 3.2 billion bases and a mass of 3 pg per haploid genome [47]. When estimating MB count for the mPCR assay, the 3 cycles of PCR used in the 1st round of the reaction and the fraction of the product used to seed the 2nd reaction were accounted for by dividing by 3.2 to give an “adjusted MB count” [48]. For error correction, reads were further processed to identify single-molecule sequences (smSequences). These are defined as the microsatellite sequences representing the majority of reads in each group that share the same MB, and are assumed to represent the original template molecules (see Supporting Information in [32]).

Sample MSI classification used a naïve Bayesian algorithm (for details, see [46]) trained using a cohort of CRC samples of known MSI status (MSS or MSI-H). The classifier analyzes the relative frequency of microsatellite deletions in each marker and the allelic distribution of these deletions in markers where linked SNPs are heterozygous. Data from both are combined into a single MSI score, with a score >0 indicating a higher probability the sample is MSI-H and a score <0 indicating a higher probability the sample is MSS.

Statistical analyses of mPCR and MIP amplification efficiencies and error rates were performed with the Wilcoxon signed-rank test, using values paired by sample and marker. The contribution of tumour DNA within preop urine and vaginal swab samples was estimated for each marker using the reference allele frequency (RAF) detected in control tissue (RAF[Control]), tumour (RAF[Tumour]), and preop samples (RAF[Preop]) and the following equation:

(RAF[Control] × N) + (RAF[Tumour] × T) = (RAF[Preop cfDNA])

where T + N = 1, N = the proportion of cfDNA derived from normal tissues, and T = the proportion of cfDNA derived from tumour.

## 3. Results

### 3.1. mPCR Classifies CRCs with 96–100% Sensitivity and Specificity

**Figure 1:**
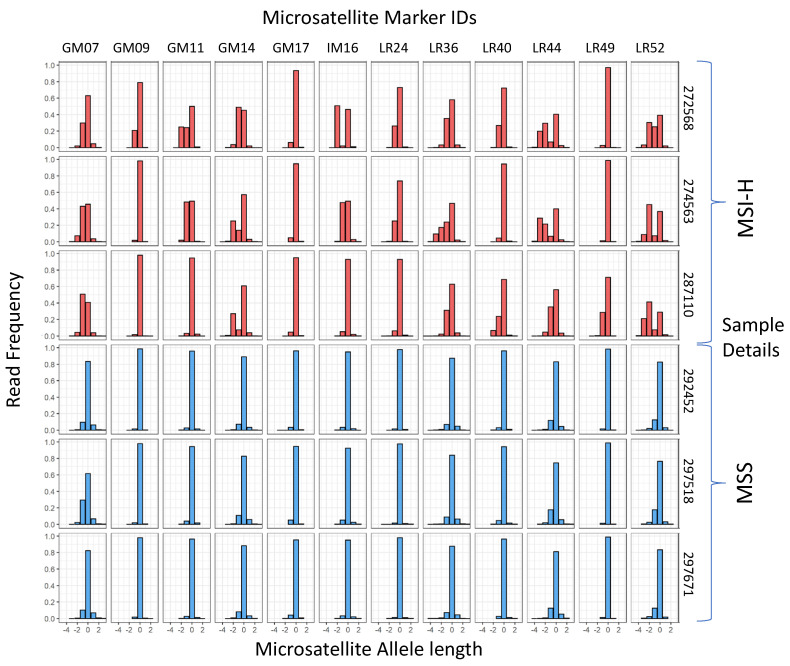
Microsatelllite allele length frequencies in training samples with known MSI status. The reference allele length is 0 for each marker. Sample classification, marker names, and sample numbers are shown.

A cohort of 100 CRCs (50 MSI-H/50 MSS), previously classified with the MSI Analysis System V1.2 (Promega, Madison, WI, USA) and the original 24-marker MIP assay [31] were analysed with the 12-marker multiplex (for details of assay redesign, see Methods and Figures S1–S4). Examples of marker read length distributions in MSI-H and MSS samples are shown in Figure 1. A high frequency of non-reference alleles can be seen in multiple markers in all 3 MSI-H samples shown.

**Figure 2:**
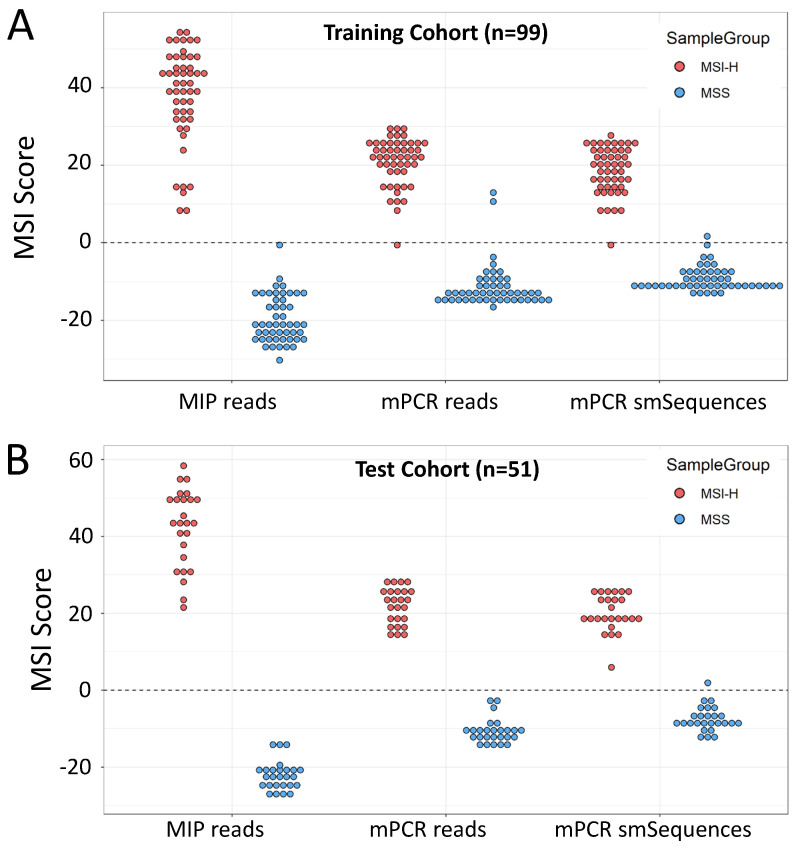
Training and validation of 12-marker mPCR assay. (**A**). Self-classification of training cohort (*n =* 99) using the 24-marker MIP and 12-marker mPCR panels. MIP amplification of the 24-marker panel is included as the gold standard comparator (**B**). MSI scores in the test cohort (*n =* 51) using classifiers trained on reads from the amplification method and read groups indicated. Scores >0 are classified as MSI-H, scores <0 as MSS.

Using MSI Analysis System V1.2 results as the reference, the MSI classifier [46] was retrained for each of 3 sequence datasets: reads from the original 24 MIP amplified microsatellites, mPCR amplicon reads, and mPCR amplicon smSequences (see Methods). One sample from the cohort gave discordant SNP alleles, indicating a sampling error, so was excluded prior to analysis. Classification using MIP reads achieved 100% sensitivity (50/50 MSI-H correctly classified) and 100% specificity (49/49 MSS correctly classified), consistent with results using the original 24-marker classifier [31], mPCR read-based classification achieved 98% sensitivity (49/50) and 96% specificity (47/49), and mPCR smSequence-based classification achieved 98% sensitivity (49/50) and 98% specificity (48/49) (Figure 2A).

The trained classifiers were then used to analyse a further cohort of 51 CRCs with known MSI status. Both MIP and mPCR read-based classification gave 100% sensitivity and 100% specificity, with mPCR smSequence-based classification giving 100% sensitivity and 96% specificity (Figure 2B) relative to the reference method (Promega). *BRAF* c.1799T > A (p.V600E) status had previously been obtained for 67 of the samples across both cohorts using high-resolution melt curve (HRM) analysis [49]. Of these, 66 were correctly called by the mPCR assay (amplicon reads), giving an overall concordance of 98.5% between HRM and mPCR. Furthermore, VAFs obtained with MIPs and mPCR were strongly correlated (R = 0.995, *p* < 2 × 10^−16^, Figure S5).

### 3.2. mPCR Identifies MSI in Individual and Composite CRC Endoscopic Biopsies from LS Patients

Knowledge of CRC MSI status prior to surgery can influence treatment options and enhance existing LS screening pathways. However, endoscopic biopsies can vary extensively in histology and tumour cell content. To establish if mPCR could be used to detect MSI in endoscopic biopsies prior to surgery, biopsies from LS patients with CRC were identified by reviewing CRC patient data between 1 April 2016 and 31 January 2021 in the Northumbria Health Care NHS Foundation Trust (see Methods). Thirty-seven patients presented with an MSI-H and *BRAF* c.1799T > A (p.V600E)-negative tumour. Of these, 6 had LS, and biopsy blocks from 4 were able to be accessed. As multiple biopsies from each patient are routinely mounted in a single FFPE block, composite tissue curls were taken by cutting across all biopsies, in addition to separate curls from each of the 19 independent biopsies (Figure S6). DNA was successfully extracted from 4/4 composite and 18/19 individual biopsies, providing 2–40 ng of template for mPCR. Curls from all 4 resected tumours were also obtained.

**Figure 3:**
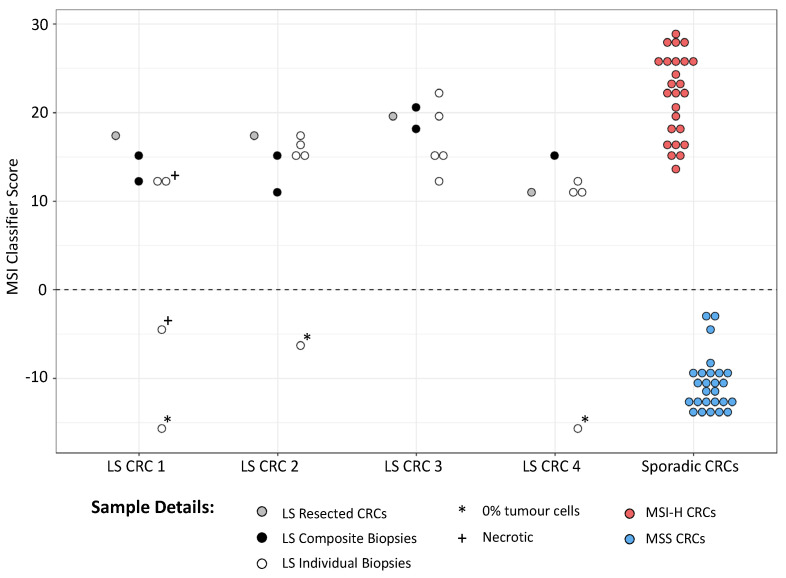
MSI classification of CRC samples from LS patients. MSI scores from CRCs, associated endoscopic biopsies, and composite biopsies from 4 LS patients are shown, along with scores from MSI-H and MSS tumours from the test cohort. Scores >0 are classified as MSI-H, scores <0 as MSS.

All samples were sequenced as before, yielding an adjusted median MB count of ~1300 per marker (minimum 249), indicating that DNA quantity was not limiting (Table S2). Given the reduced sensitivity and specificity obtained with mPCR smSequence data in the test cohort (Figure 2), classification was based on amplicon reads only. All composite biopsies and 14/18 individual biopsies were classified as MSI-H, with assay scores similar to those from the patient resected tumours and MSI-H CRCs from the test cohort (Figure 3). Of the 4 MSS biopsies, 3 were found to have no tumour cells upon pathology analysis, and the 4th was 1 of 2 necrotic biopsies where tumour cell content could not be assessed. All other biopsy samples had a minimum of 20% tumour cell content, apart from 1 where only dysplasia was observed (Table S2).

### 3.3. Case 1: mPCR Can Detect UTUC-Derived MSI-H cfDNA in Urine from an LS Patient

**Figure 4:**
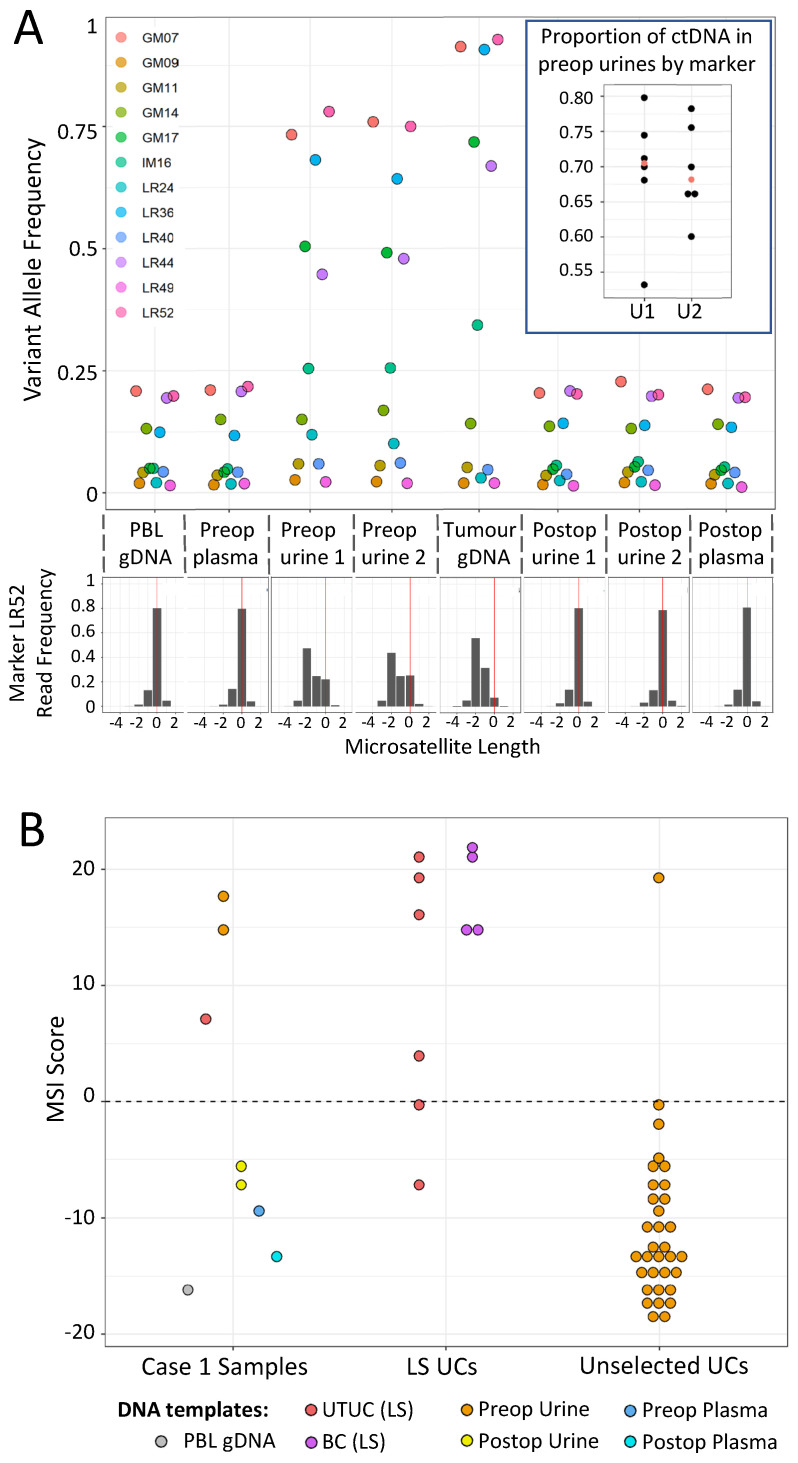
Analysis of Case 1 (LS UTUC patient) samples with the mPCR assay. (**A**) *Top*—VAFs for all 12 markers in patient samples (key gives marker names). Inset—Dot plot showing proportion of tumour-derived DNA within preop urine samples, based on 6 markers with increased VAFs. The median value is shown in red. *Bottom*—example of relative read frequencies of microsatellite lengths for 1 marker, LR52 (reference allele length = 0). (**B**) MSI scores of samples from Case 1 (left), UCs from genetically confirmed LS patients (centre), and preop urine samples from unselected UC patients (right). For details of samples, MSI scores, and read/barcode numbers, see Table S3.

UTUC is the 3rd most common LS tumour, but no accurate surveillance method is available to LS gene carriers. As a proof of concept for urine-based detection of UTUCs in LS patients using mPCR, samples were analysed from a 68-year-old male with a deletion of *MSH2* exon 3 and a confirmed UTUC (Case 1). The patient presented with loin pain and haematuria, and imaging revealed a 4 cm tumour of the right renal pelvis. Histological analysis following a right nephroureterectomy, showed high-grade papillary transitional cell carcinoma invading the peripelvic fat. Preop and postop urine cfDNA were analysed, together with DNA samples from their PBLs, preop plasma, and resected tumour. In the patient’s 2 preop urine cfDNA samples, 6 out of 12 MSI markers exhibited elevated VAFs relative to both PBL and postop urine DNAs (Figure 4A), and all 6 had even higher VAFs within the resected tumour. No increase in VAF was observed in preop plasma. This is consistent with the elevated VAFs in the urine cfDNA being due to tumour DNA, and subsequent analysis of VAFs within tumour and urine samples suggested that approximately 70% of the cfDNA in the urine was tumour-derived (Figure 4A inset and see Methods). Furthermore, the preop urine and resected tumour samples were all classified as MSI-H, while the PBL, post op, and preop plasma samples classified as MSS (Figure 4B). We also analysed UTUC and bladder urothelial cancer (BUC) samples from genetically confirmed LS patients, together with preop urine samples from a cohort of unselected UC patients. These represented control populations where the frequency of MSI-H tumours were known to be high and low, respectively, as the estimated MMRd frequency in LS UCs is 85.0% (95% CI: 69.3–96.2%), compared to only 3.5% (95% CI: 2.5–4.7%) in unselected UCs [29]. Consistent with expectations, 8 out of 10 urinary tract tumours from LS patients were MSI-H, compared to only 1 MSI-H tumour among the 33 unselected UC patient urines (Figure 4B).

### 3.4. Case 2: mPCR Can Detect EC-Derived MSI-H cfDNA in Urine from an LS Patient

**Figure 5:**
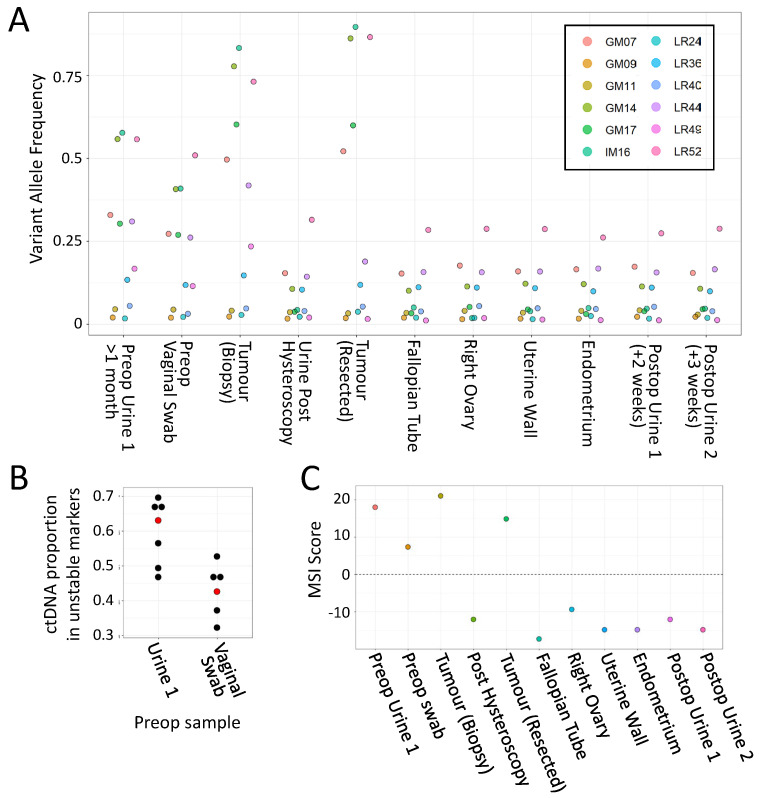
Analysis of Case 2 (LS EC patient) samples with the mPCR assay. (**A**) VAFs for all 12 markers in Case 2 samples. (key gives marker names) (**B**). Estimates of proportion of tumour-derived DNA within preop samples, based on 6 markers with increased VAFs. Median values are shown in red. (**C**) MSI classifier scores of all Case 2 samples. For details of samples, classifier scores, read/barcode numbers, and examples of marker allele distributions, see Table S3 and Figure S7.

Finally, while preparing the manuscript, a former patient with an *MLH1* pathogenic splice variant (c.588 + 1G > A) contacted the clinical team for assistance, as over several months they had experienced severe dysfunctional menstrual bleeding (Case 2). Ultrasound revealed a likely benign fibroid, but, aware of the high risk of EC, the patient sought advice and arranged a private consultation with a gynaecologist. The clinical team requested MSI analysis of a urine sample and a vaginal swab to support the case for more urgent investigation and consideration of hysterectomy. A clear MSI signal was detected in both, with 6 markers having very high VAFs (Figure 5A, lanes 1 and 2). An urgent referral for hysteroscopic biopsy was arranged, which revealed a high-grade endometrial adenocarcinoma with heterogeneous morphology, the bulk of which was removed by curettage. MMR immunohistochemistry showed loss of nuclear staining for MLH1 and PMS2. Fresh tumour tissue from the hysteroscopic biopsy specimen showed even higher VAFs in the same 6 markers (Figure 5A, lane 3). Once symptoms had subsided after hysteroscopy, the patient took a further urine sample, which they delivered by post, where only a single marker had a VAF >0.25 (LR52, Figure 5A, lane 4).

**Figure 6:**
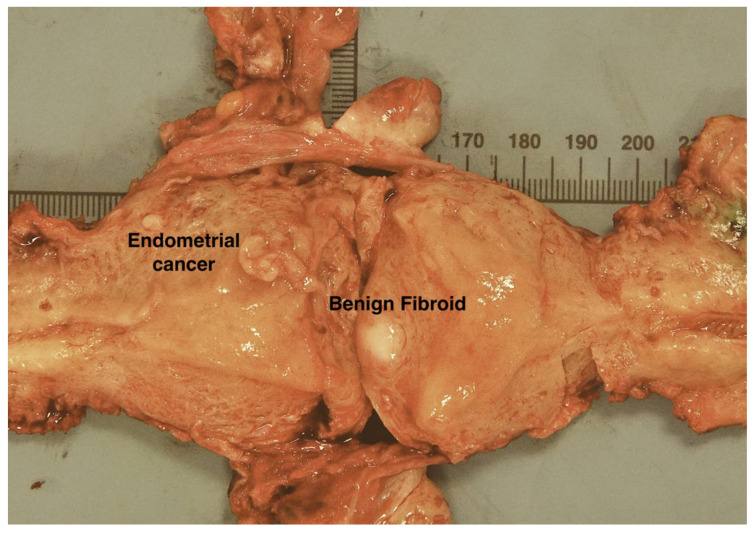
Case 2. Uterus bisected along its lateral walls showing a fleshy polypoid endometrial carcinoma arising from the left lateral fundus, in the region of the left cornua. The bulk of the tumour was removed during diagnostic hysteroscopic endometrial curettage. Scale is in mm.

Nine days later, they underwent a hysterectomy and bilateral salpingo-oophorectomy, which identified a polypoid tumour in the region of the fundus towards the left lateral cornua, and a benign leiomyoma within the uterine myometrium (Figure 6). MSI analysis of the tumour found that 5 markers out of the 6 unstable in urine had even higher VAFs than the biopsy (>50%, Figure 5A lane 5). In contrast, only a single marker (LR52) had a VAF >0.25 in non-neoplastic resected tissues and postop urines (Figure 5A, lanes 6–11).

Collectively, these results are consistent with the preop urine MSI signal being from the tumour, and subsequent analysis of VAFs within the preop, postop, and tumour biopsy samples estimated that over 60% of the cfDNA in the initial preop urine was tumour-derived (Figure 5B). In addition, only the first preop urine, vaginal swab, and tumour samples were classified as MSI-H, with all other urine samples and control tissues classifying as MSS (Figure 5C).

## 4. Discussion

We have shown that an mPCR amplicon sequencing assay can classify CRC MSI status and detect *BRAF* variants with a high level of concordance relative to existing diagnostic methods, and that it can be used to detect MSI in colonoscopic biopsies.

Knowledge of biopsy MMR status could facilitate clinical decision making and LS diagnosis, and establish the MMR status of tumours where surgery is not performed. Our original MIP assay classified CRCs with as little as 3% MSI-H template in mixing experiments [31], and composite biopsy templates analysed here gave assay scores comparable to matched resected tumour samples chosen via pathology triage for high tumour cell content. Composite samples could, therefore, potentially be used for MSI analysis without prior assessment. The method could also enable expansion of LS screening to adenomas removed during routine colonoscopic investigation, as MMRd/MSI often precedes adenoma formation in LS patients (e.g., [50,51,52]), and it has been estimated that 69.5% of conventional adenomas in LS patients are MMRd/MSI-H [53]. However, data from only 4 LS patients have been analysed here, so it will be important to investigate adenomas and biopsies from larger cohorts of patients and to include tumours of both sporadic and inherited origins.

We also detected strong MSI signals within urine cfDNAs from both a UTUC and an EC patient, consistent with signals subsequently found in their resected tumours. This could have important implications for preop detection of MMR deficiency in these tumours in the general population and for tumour surveillance in LS.

UTUC is the 3rd most frequent tumour type in the LS spectrum [35] and is particularly common in patients with defects in *MSH2*, but while European guidelines for LS patient management acknowledge that surveillance for early detection of this tumour is clinically desirable, there are no cost-effective non-invasive techniques routinely available [54]. Both amplicon sequencing [55] and target-capture/deep-sequencing approaches [48] have been used to identify tumour DNA within urine cfDNA at the point of diagnosis, and capture approaches have since been adapted to incorporate MSI markers (e.g., [56,57,58]). ddPCR-based MSI assays [59,60] have also recently been developed that are potentially simpler and more cost-effective options for early detection of LS-derived tumours. Here, mPCR identified a clear MSI signal from an LS patient’s UTUC prior to surgery, with approximately 70% of preop cfDNA being tumour-derived. While this appears high, it is not exceptional; the quantity of cfDNA within urine per se has been proposed as a biomarker for bladder cancer (BC) [61], urinary tumour DNA levels from fragment capture/sequencing of BCs can exceed 40% in samples taken at diagnosis [48], and can be high enough in urine (or urinary sediment) that microsatellite markers have been used to detect copy-number imbalances ([62,63,64,65]; reviewed in [66]). Subsequent to our analysis, the patient also underwent surgery for a pancreatic adenocarcinoma aged 68, a small bowel gastrointestinal stromal tumour aged 69, and a bladder urothelial cancer aged 70. However, we have yet to analyse these malignancies for MSI.

We also detected an MSI signal in preop voided urine samples and vaginal swabs from an LS patient with a suspected EC. The absence of a signal in postop urine indicates that the EC is the source rather than, for example, an undiagnosed UTUC. The proportion of tumour-derived DNA in the initial urine and vaginal swab samples was again high (>60% and >40%, respectively) and is consistent with existing evidence that material from gynaecological cancers frequently contaminates the lower urinary tract [37,38,39]. The absence of an MSI signal in the urine sample taken after hysteroscopy is also noteworthy. Removal of much of the tumour by curettage could account for this, but clearly visible polypoid tumour material remained (Figure 6). Thus, while our results illustrate that there can be significant contamination of urine with tumour-derived cfDNA, it can also be variable, suggesting that the timing of preop urine collection in relation to symptoms and other factors will require detailed investigation.

While these results highlight the potential of MSI analysis of urine as a cheap routine surveillance tool, an obvious caveat is that only a single tumour of each type has been analysed, effectively representing proof-of-principle experiments. Much larger numbers of UC and EC samples from LS patients will need to be analysed, and/or patient groups at increased risk of developing these cancers, such as *MSH2*-variant carriers for UTUCs [2] and patients presenting with post-menopausal bleeding for EC [37]. In addition, the mPCR assay would benefit from further refinement, as the lower limit of detection (LLoD) is likely to be similar to the parent assay (3% tumour DNA [31]), much higher than the LLoD of both capture/deep sequencing (e.g., ~0.4% [58]) and ddPCR (~0.2%, [60]). This could be improved by using more sophisticated data analysis methods (e.g., [32]), using a high-fidelity polymerase in the first round of PCR, and by increasing marker read depth to enable analysis of smSequences rather than raw reads. Mutation hotspots in such genes as *FGFR3*, *TERT*, *PLEKHS1*, and *GPR26*, frequently mutated in UTUCs [55,67,68], or the coding regions of such genes as *PTEN* and *PIK3CA*, frequently mutated in EC [69,70], could also be incorporated into the multiplex to identify MSS tumours that, although in the minority, do occur within LS patients.

## 5. Conclusions

We have demonstrated that an mPCR amplicon sequencing assay can detect MSI within the 3 most common LS tumour types using biopsy or urine samples taken prior to surgery. The low cost and scalability of the assay [31] could help to meet the increased demands for assessment of tumour MMR/MSI status by increasing the range of samples which can be tested routinely. For instance, as the frequency of LS among patients with EC and UTUC is comparable or higher than in CRC [71,72,73], routine MSI analysis of urine prior to tumour resection could enhance LS screening, as well as provide information for disease management. With further adaptation, the assay could also enable routine, postal-based, liquid biopsy-based surveillance of LS carriers to improve detection and outcome in urinary tract and gynaecological cancers.

## Data Availability

All sequence data have been submitted to the European Nucleotide Archive under study number PRJEB52735.

## Supplementary Materials

The following supporting information can be downloaded at: https://www.mdpi.com/article/10.3390/cancers14153838/s1, Figure S1: Comparison of MIP and 12-marker mPCR amplification; Figure S2: Representation of markers within MIP and mPCR assay; Figure S3: mPCR MB counts in template dilutions; Figure S4: Estimates of baseline sequence length error rates in MIP and mPCR assays; Figure S5: Correlation between MIP and mPCR *BRAF* c;1779T variant allele frequencies; Figure S6: CRC biopsy samples and mPCR amplification; Figure S7: Marker allele length frequencies in EC patient samples; Table S1: mPCR primers, sequencing primers, and MIP probes; Table S2: CRC biopsy barcode counts and classification; Table S3: Barcode counts and classification of UTUC and EC samples.
