# Supplementary material for: Detection of Microsatellite Instability in Colonoscopic Biopsies and Postal Urine Samples from Lynch Syndrome Cancer Patients Using a Multiplex PCR Assay
Source: Cancers (Basel). 2022 Aug 8;14(15):3838. doi: 10.3390/cancers14153838 (PMC9367254; doi:10.3390/cancers14153838)
Supplement: Supplementary file 1 [file cancers-14-03838-s001.zip › cancers-1773862-supplementary figures.pdf]

cancers

MDPI

Supplementary Materials

Detection of Microsatellite Instability in Colonoscopic Biopsies and Postal Urine Samples from Lynch Syndrome Cancer Patients Using a Multiplex PCR Assay

Rachel Phelps, Richard Gallon, Christine Hayes, Eli Glover, Philip Gibson, Ibrahim Edidi, Tom Lee, Sarah Mills, Adam Shaw, Rakesh Heer, Angela Ralte, Ciaron McAnulty, Mauro Santibanez-Koref, John Burn and Michael S. Jackson

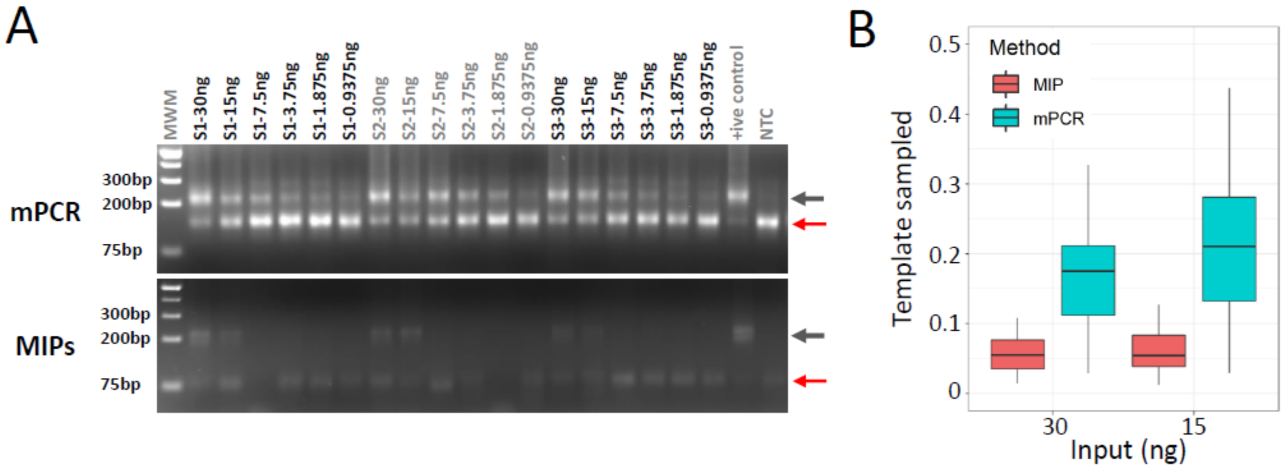

Figure S1. Comparison of MIP and 12 marker mPCR assay. (**A**) Control PBL template dilution series. Amplicons were successfully generated in triplicate from all template quantities using mPCR, but the MIP-based assay only generated clear amplicons from the 30 ng and 15 ng templates. Amplicons are indicated with black arrows, primer dimers indicated with red arrows. MWM = Molecular Weight Marker, NTC = No template control. Sequence data was generated from these for analysis (see methods). (**B**) Fraction of template sampled at 30 ng and 15 ng input. Box and Whisker plot where box encompasses upper and lower quartiles, median is indicated by a horizontal line, and outliers are shown as solid circles. Across all 12 markers, the mPCR assay sampled a higher proportion of template DNA molecules than the MIP assay [17.5% v 5.5% at 30 ng and 21% v 3.8% at 15 ng ($p = 9.6 \times 10^{-10}$ and $p = 5.5 \times 10^{-10}$ respectively)]. The proportion of template molecules sampled was estimated assuming a C-value of 3.2 billion bases and a mass of 3pg per haploid genome [47].

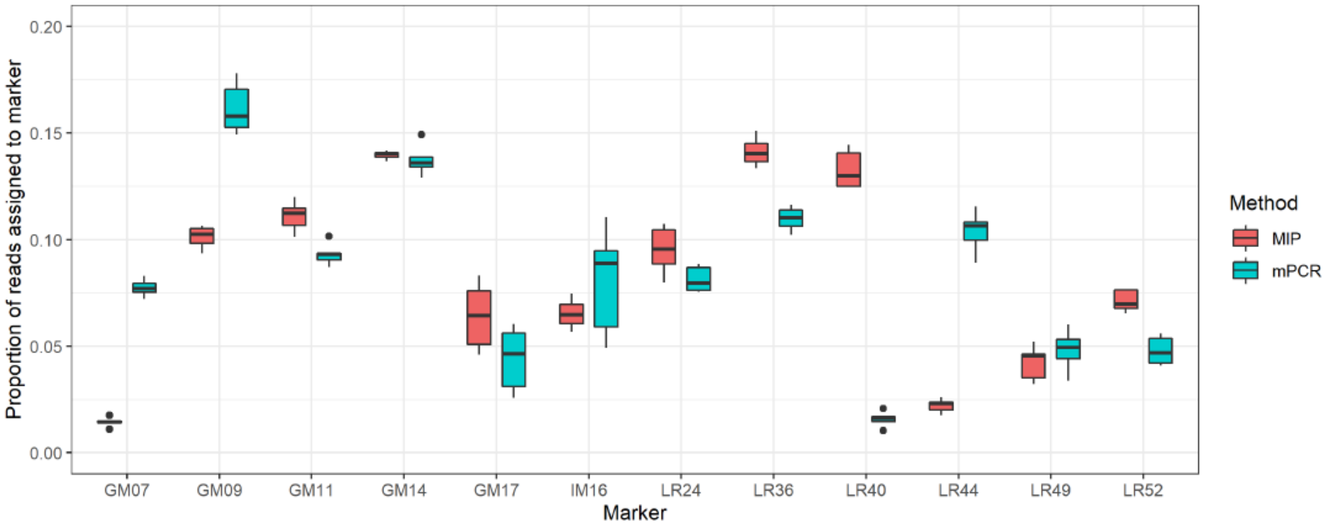

Figure S2. Representation of markers within MIP and mPCR assay. Box and Whisker plot where box encompasses upper and lower quartiles, median is indicated by a horizontal line, and outliers are shown as solid circles.

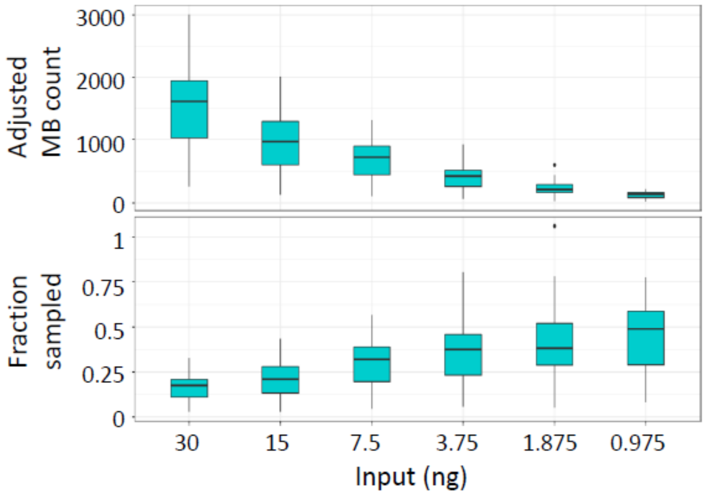

Figure S3. mPCR MB counts in template dilutions. **Top panel**—Median number of MBs recovered by mPCR per marker. The estimated number of unique template molecules sampled fell from over 1500 (30 ng), to 142 (~1 ng). MB count was adjusted to account for 3 rounds of barcode incorporation (see methods). **Lower panel**—fraction of template molecules sampled as estimate of reaction efficiency. As template quantity decreased, the proportion of available template molecules that were sequenced increased from 17.5% to ~50%.

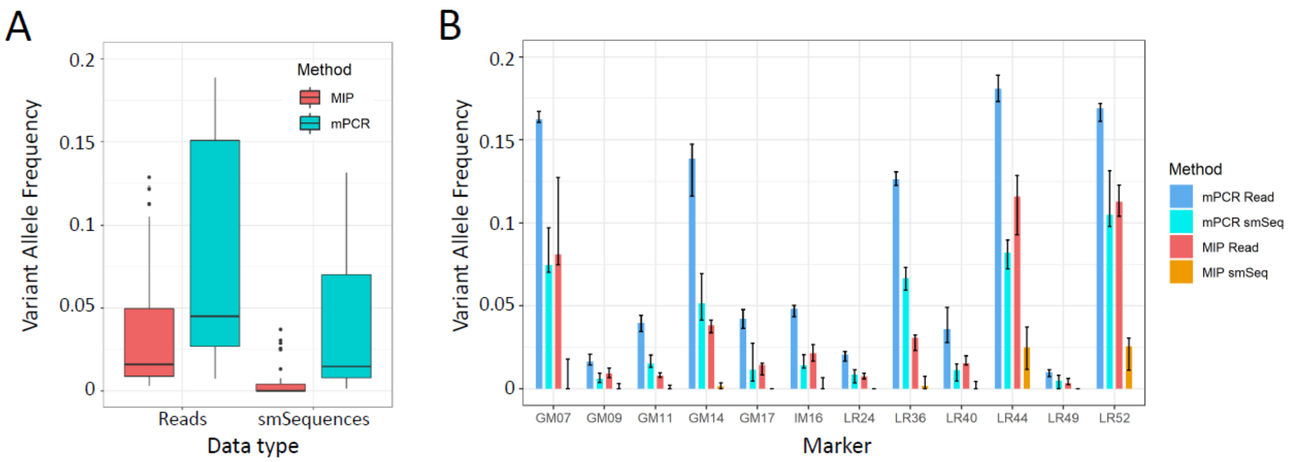

Figure S4. Estimates of baseline sequence length error rates in MIP and mPCR assays. Error rates could be affected by use a polymerase with no 3′–5′ proofreading function in the 1st 3 cycles of the mPCR assay. Error rates were estimated from microsatellite variant allele frequencies (VAFs) in control PBL DNAs (30 ng and 15 ng templates), both before and after error correction using smSequences (see Methods), on the assumption that no microsatellite length variation is present prior to amplification. (**A**) Summary data. Box and Whisker plot where box encompasses upper and lower quartiles, median is indicated by a horizontal line, and outliers are shown as solid circles. Error rates were significantly higher in the mPCR assay for both read (4.50% v 1.61%, $p = 2.91 \times 10^{-11}$) and smSequence (1.47% v < 0.001%, $p = 2.91 \times 10^{-11}$). (**B**) Marker specific error rates.

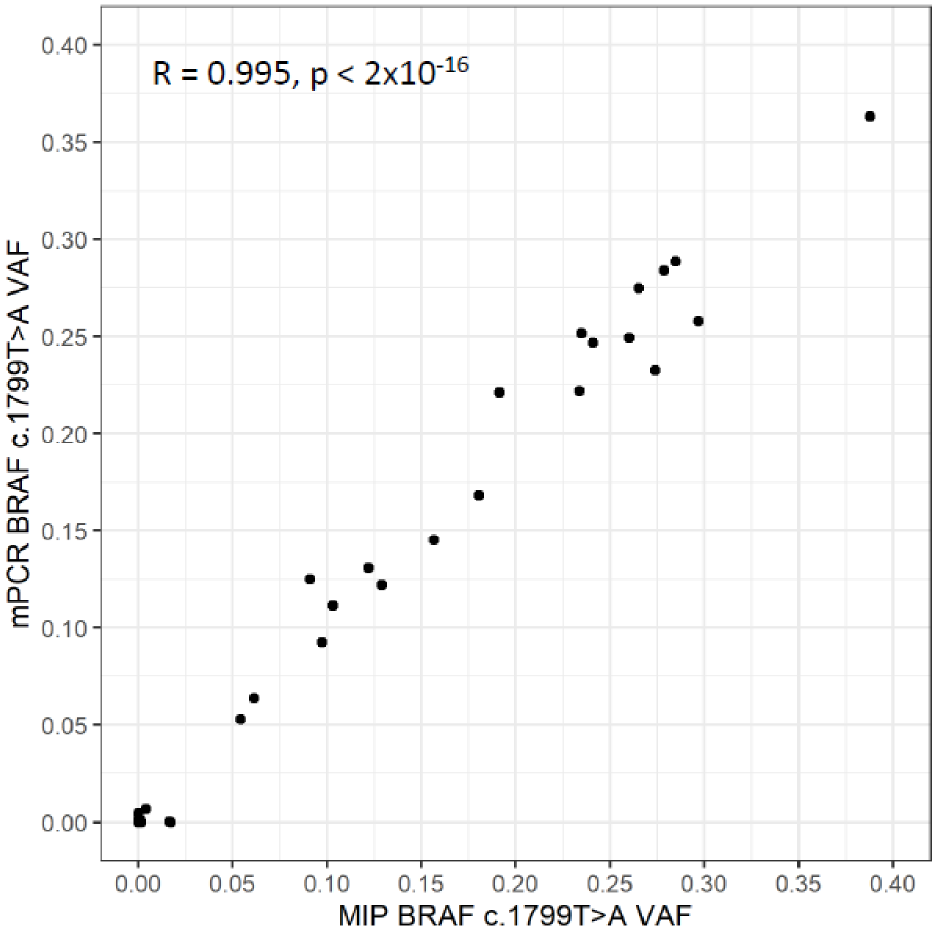

Figure S5. Correlation between MIP and mPCR BRAF c.1779T variant allele frequencies. Data is from 107 samples in the training and test cohorts analysed using both MIP and mPCR methods.

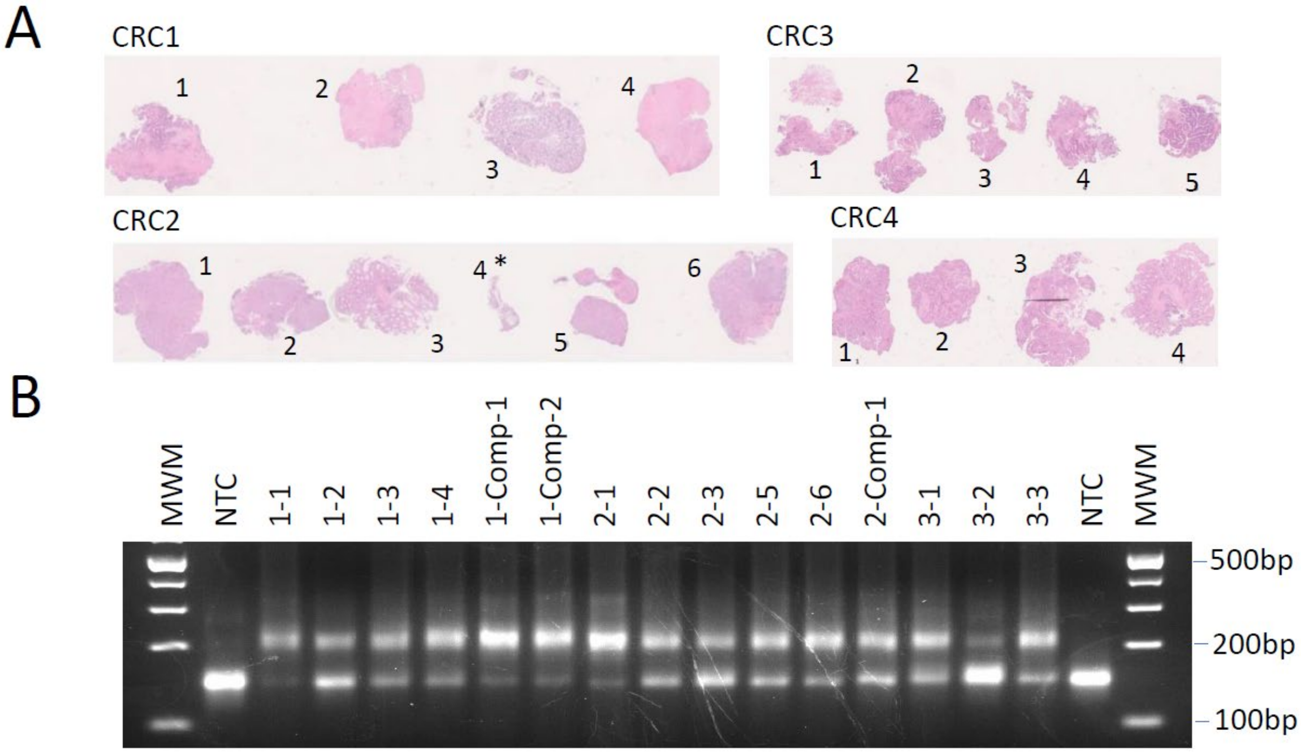

Figure S6. CRC biopsy samples and mPCR amplification. (**A**) H+E stained images of FFPE paraffin embedded biopsies from each patient, with each numbered relative to amplicons and data generated. (**B**) Example of mPCR amplicons (>200 bp) and primer dimers (<200 bp) from individual and composite biopsies. NTC = No template control. MWM = Molecular weight marker.

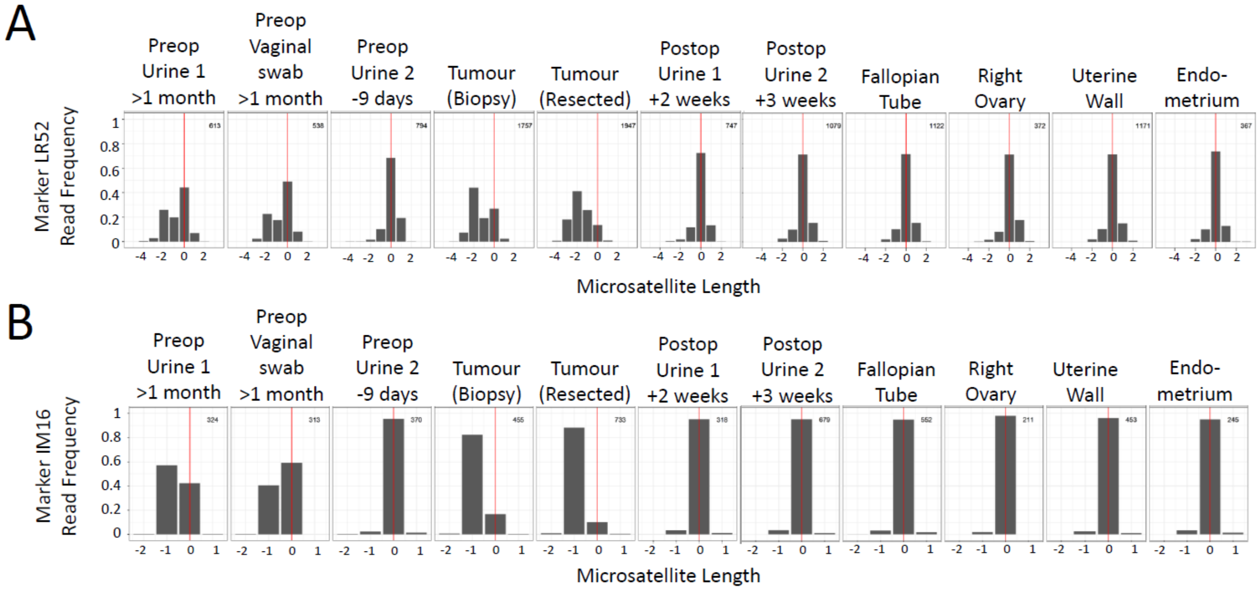

Figure S7. Marker allele length frequencies in EC patient samples. (**A**) LR52; wild type allele is reduced and smaller alleles (−1 to −4) are enriched in preop (>1 month) and tumour samples. (**B**) IM16; wild type allele is reduced and −1 allele is enriched in preop (>1 month) and tumour samples only. The reference allele is 0, highlighted with a red line. Numbers indicate reads contributing to each plot.
